# Changes in Sleep Quality and Insomnia Severity After Day Therapy in Patients with Alcohol Dependence: A Before-and-After Case Series

**DOI:** 10.3390/jcm15041400

**Published:** 2026-02-11

**Authors:** Wolińska Weronika, Giezek Marta, Szylińska Aleksandra, Scech Marcin, Anna Knyszyńska

**Affiliations:** 1 Independent Laboratory of Humanities and Occupational Therapy, Pomeranian Medical University in Szczecin, 71-103 Szczecin, Poland; marcin.scech@pum.edu.pl (S.M.); anna.knyszynska@pum.edu.pl (A.K.); 2 Subdepartment of Social Medicine and Public Health, Department of Social Medicine, Pomeranian Medical University in Szczecin, 71-210 Szczecin, Poland; marta.giezek@pum.edu.pl; 3 Department of Cardiac Surgery, Pomeranian Medical University in Szczecin, 70-111 Szczecin, Poland; aleksandra.szylinska@pum.edu.pl

**Keywords:** addiction, alcohol, insomnia, sleep quality, daytime therapy

## Abstract

**Background/Objectives:** Alcohol use disorders (AUDs) and heavy alcohol consumption remain major global public health concerns, substantially increasing morbidity, mortality, and the overall burden of disease. Insomnia affects 15–30% of the general population and up to 36–91% of individuals with AUD, and evidence suggests a bidirectional relationship in which sleep disturbances may contribute to future alcohol consumption. The primary objective of the study was to assess the impact of an eight-week day therapy program on sleep quality and the severity of insomnia symptoms in individuals with alcohol dependence. **Methods:** The survey was conducted at the Addiction and Co-Addiction Therapy Center in northwestern Poland. Patients completed the survey questionnaires during individual meetings with a researcher, who personally administered the survey both before and after therapy. Ninety-five patients participated in the study, including 32 women (33.68%) and 63 men (66.32%). The study used the Athens Insomnia Scale (AIS) and the Pittsburgh Sleep Quality Questionnaire (PSQI), as well as a questionnaire containing sociodemographic data. **Results:** Before treatment, the mean AIS score for the entire sample was 9.09 ± 5.22, indicating clinically significant insomnia. After 8 weeks of therapy, the AIS score decreased to 5.22 ± 3.33. Similarly, the mean total PSQI score declined from 7.12 ± 3.99 at baseline to 4.13 ± 3.33 following treatment, reflecting a significant improvement in sleep quality. **Conclusions:** Eight weeks of daytime therapy significantly reduced insomnia severity in alcohol-dependent individuals, with a >40% decrease in AIS scores and a marked improvement in sleep quality reflected by a reduction in PSQI scores to the cutoff level.

## 1. Introduction

Alcohol use disorders (AUDs) and heavy alcohol consumption remain common public health problems worldwide. Alcohol consumption has a complex connection with health. Scientists have identified alcohol consumption as a major risk for disease burden, and studies have linked alcohol consumption to 60 acute and chronic diseases, including oropharyngeal and esophageal cancer, liver cancer, breast cancer, depression, epilepsy, alcohol use disorders, hypertension, hemorrhagic stroke, and liver cirrhosis [1,2]. In this area, we should distinguish excessive alcohol consumption, which is defined by specific consumption thresholds (e.g., ≥4/5 drinks on the same occasion for women/men, respectively), and alcohol use disorder, i.e., AUD. It is a chronic and relapsing disorder, affecting individuals who are unable to control or stop drinking alcohol, despite negative consequences (e.g., interpersonal or work-related problems). With the introduction of the DSM-5 criteria, the concepts of alcohol abuse and dependence were replaced by alcohol use disorder (AUD). The diagnosis of AUD is based on 11 diagnostic criteria: all criteria for alcohol dependence, three criteria for alcohol abuse, and alcohol craving [3]. Additionally, AUD is described as a chronic recurrent disorder [4]. Both AUD and heavy alcohol consumption increase the risk of adverse health consequences, contributing to high mortality and the global burden of disease. The consequences of harmful alcohol use are observed not only in individual health but also in terms of social and economic losses for the entire country. Therefore, preventing and treating AUD is an ongoing public health challenge [5]. Data indicating the impact of harmful alcohol consumption on premature death and disability are particularly concerning. According to the World Health Organization, harmful alcohol consumption is associated with approximately 3 million deaths per year, representing 5.3% of all deaths globally. This percentage increases among people aged 20–39, accounting for approximately 13.5% of all deaths in this age group [6].

Insomnia is a common complaint, affecting 15–30% of the general population [7] and as many as 36–91% of individuals with alcohol use disorder (AUD) [8,9]. Insomnia is defined in the DSM-5 as dissatisfaction with the quality or quantity of sleep due to problems initiating or maintaining sleep, resulting in daytime distress or impairment in functioning [3]. Studies have shown that in individuals treated for AUD, insomnia changed over the course of treatment. During early discontinuation, i.e., the first two weeks of treatment, insomnia symptoms occurred in 88% [10], and in the early phase, i.e., up to about eight weeks, symptoms were reported by about 65% of patients [11]. Studies have shown that insomnia is also a robust predictor of relapse [12]. The coexistence of AUD and insomnia likely has a causal relationship in which sleep problems influence future alcohol consumption in adults [13] and adolescents [14], and heavy and prolonged alcohol use may lead to insomnia [13]. Insomnia symptoms can disrupt all stages of sleep, and a study of Polish alcohol-dependent patients found that nearly 63% had delayed sleep induction, 57% frequently woke up during the night, and 41% woke up earlier than planned [15]. Insomnia symptoms improve after alcohol withdrawal. A 4-week alcohol withdrawal treatment resulted in a reduction in sleep disturbances among 20% of those treated [16]. Unfortunately, alcohol consumption is one of the most common examples of self-medication for sleep problems in the general population [17,18], while the recommended treatment for insomnia is cognitive-behavioral therapy (CBT-I). Interestingly, previous meta-analyses of studies and systematic reviews in this area have shown a moderate effect of cognitive-behavioral therapy on alcohol-dependent patients struggling with insomnia [19,20]. Although sleep disturbances in alcohol use disorder have been the subject of numerous studies, little is known about how sleep quality and insomnia change during structured day hospital addiction treatment programs, which represent an intensive, non-residential form of care.

The primary objective of the study was to assess the impact of an eight-week day therapy program on sleep quality and the severity of insomnia symptoms in individuals with alcohol dependence.

## 2. Materials and Methods

### 2.1. Study Design

This before-and-after case series is reported in accordance with the CARE guidelines [21]. See Supplementary Material File S1 for CARE Checklist of information to include when writing a case report.

#### 2.1.1. Setting and Ethical Consideration

The survey was conducted between January and December 2024 at the Addiction and Co-Addiction Therapy Center in northwestern Poland. The inclusion criterion for the study was a diagnosis of alcohol addiction, attendance at the center for basic day therapy, residence in the West Pomeranian Voivodeship, and consent to participate in the study.

Approval for the study was obtained from the Bioethics Committee of the Pomeranian Medical University in Szczecin (KB-.006.29.2024).

#### 2.1.2. Participants

Patients completed the survey questionnaires during individual meetings with a researcher, who personally administered the survey both before and after therapy. Ninety-five patients participated in the study, including 32 women (33.68%) and 63 men (66.32%). The mean age of participants was 44.50 ± 11.62 years (median: 42 years; range: 23–75 years). All participants lived in an urban area in northwestern Poland. Before therapy, 30 (31.57%) patients were taking both prescription and over-the-counter sleep medications; after therapy, 26 (27.36%) were taking such medications. Of the participants, 45 (47.36%) reported sleeping alone in their room.

Treatment in the day hospital is a guaranteed service financed from public funds under an agreement with the National Health Fund and is available to patients free of charge. The therapeutic program lasts eight weeks and is conducted in an open group format, allowing new participants to join at any time during the cycle. The rotation of group composition promotes learning through observation and the exchange of experiences—patients beginning therapy have the opportunity to benefit from the resources and knowledge of those completing the treatment process.

Therapeutic interventions are based on an integrative approach, combining elements of cognitive-behavioral therapy, humanistic therapy, and motivational interviewing. The therapeutic model involves strengthening the patient’s autonomy, developing intrapsychic and interpersonal competencies, and developing the ability to maintain abstinence by modifying dysfunctional behavioral patterns and emotional regulation standards. A key element is to create a therapeutic environment that supports repair processes, enabling ongoing changes in daily functioning.

The program is multidimensional and adapted to the individual and group needs of its participants. It includes psychoeducation on the mechanisms of addiction and recovery, workshops on developing skills for coping with stress, anxiety, shame, and anger, interpersonal communication training, assertiveness classes, as well as relaxation techniques and elements of mindfulness training. Patients also benefit from psychiatric consultations, and in justified cases, sick leave is available for the duration of therapy. The intensity of the interventions is comparable to the one offered in inpatient hospital units, while maintaining the ability to function in a home environment and gradually implement acquired skills. Completion of the basic program prepares patients for further treatment in the form of in-depth therapy in an outpatient setting. The integrative approach employed, combining elements of cognitive-behavioral therapy, humanistic therapy, and motivational interviewing, reflects a model of addiction psychotherapy commonly used in Poland. Many public and private centers utilize a range of techniques and interventions drawn from various therapeutic approaches, tailored to the complex clinical needs of patients. Therefore, the results obtained can be considered broadly representative of clinical practice implemented in similar programs in other regions of the country.

#### 2.1.3. Data Sources

The study used the Athens Insomnia Scale (AIS) and the Pittsburgh Sleep Quality Questionnaire, as well as a questionnaire containing sociodemographic data.

The Athens Insomnia Scale (AIS) consists of 8 questions, 5 of which concern nocturnal sleep and 3 of which concern daytime functioning over the past month. Responses are scored from 0 (no difficulty) to 3 (severe difficulty). The score is the sum of the obtained points and ranges from 0 to 24, where 0–5 points indicate no insomnia, 6–10 points are the cutoff value, and a score higher than 10 points indicates insomnia [22].

The Pittsburgh Sleep Quality Index (PSQI) is a standardized, quantitative tool for assessing sleep quality over the past month. Results demonstrate high levels of reliability, validity, and consistency. Due to its relatively short duration, it is frequently used in research and clinical practice. The questionnaire consists of nine questions with subitems, for a total of 19 items to be completed by the respondent. The first four questions require additional information from the respondent and address their typical bedtime, the time (in minutes) it takes to fall asleep, their usual morning wake-up time, and their average sleep duration. The next questions ask the respondent to indicate on a four-point Likert scale how often, over the past four weeks, they experienced poor sleep due to: inability to fall asleep for 30 min, waking up in the middle of the night, needing to go to the bathroom, breathing problems, coughing or snoring, feeling hot or cold, experiencing pain, or other problems they indicate. The next questions ask how often the respondent takes sleep medication, how often they have trouble concentrating, and how often they lack the energy to perform daily tasks. This section of the questionnaire uses the following response options: 0—not once in a month, 1—less than once a week, 2—once or twice a week, and 3—three or more times a week. The next question concerns self-assessment of sleep, where 0 represents very good, 1 represents fairly good, 2 represents rather poor, and 3 represents very poor. A total score ranging from 0 to 21 points is obtained by adding the scores for seven components: C1 represents subjective sleep quality; C2 represents sleep onset, which measures sleep time from fall asleep in minutes, when sleep time increased to 30 min; C3 represents sleep duration in hours; C4 represents sleep efficiency, which measures the ratio of hours of sleep to actual time spent in bed; C5 represents sleep disturbances and the impact of factors interfering with sleep, such as feeling cold or hot, pain, cough, shortness of breath; C6 represents frequency of sleep medication use; and C7 represents impairment in daily functioning. Scores are calculated for each of the seven components. Each component ranges from 0 to 3 points, with 0 representing “no difficulty,” 1 representing “slight difficulty,” 2 representing “great difficulty,” and a score of 3 representing “extreme difficulty.” You can score a total of 21 points, and the higher the score, the worse the sleep quality. A score of more than 5 points indicates reduced sleep quality and represents “poor sleepers,” while a score of less than 5 points represents “good sleepers” [23].

#### 2.1.4. Statistical Analysis

Statistical analysis was performed using licensed Statistica 13.0 software (StatSoft, Inc., Tulsa, OK, USA). The Shapiro–Wilk test was used to assess the normality of the distribution of the study variables. Quantitative data before and after therapy were analyzed using Wilcoxon Signed-Rank Test. A significance level of *p* ≤ 0.05 was adopted.

## 3. Results

### Analysis

**Figure 1:**
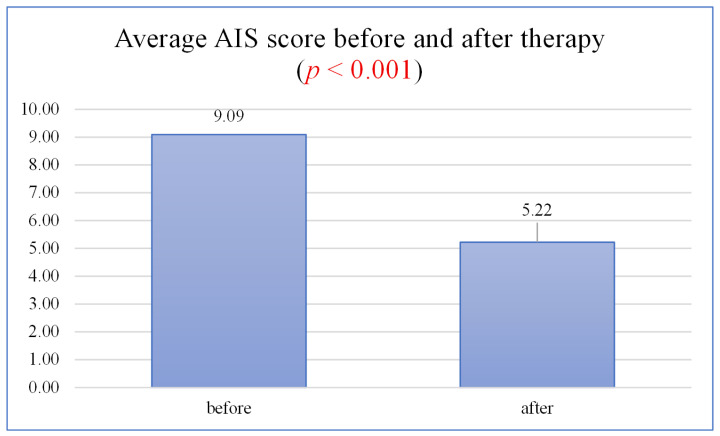
Mean AIS score before and after therapy.

The mean score obtained on the Athens Insomnia Scale (AIS) for the entire sample before treatment was 9.09 ± 5.22 points, indicating severe insomnia symptoms exceeding the commonly accepted cutoff point (≥6 points). After 8 weeks of treatment, the mean score decreased to 5.22 ± 3.33 points (*p* < 0.001)—Figure 1. This indicates a significant reduction in insomnia severity. In clinical practice, this result may indicate that many patients have moved from the area of clinical insomnia to a zone below the diagnostic threshold. This change is not only statistically significant but also clinically significant, indicating a real improvement in symptoms.

**Figure 2:**
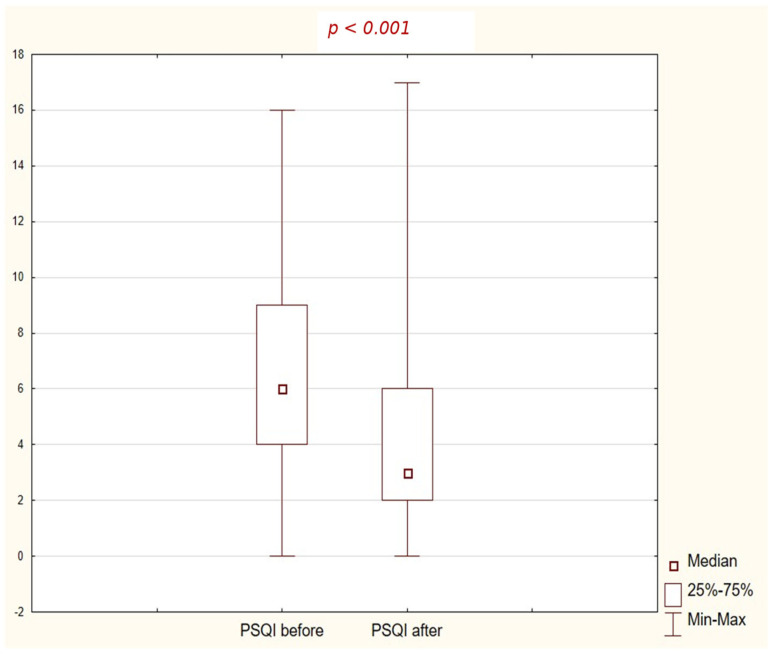
Detailed component analysis identified which aspects of sleep quality improved the most.

The total PSQI score before therapy averaged 7.12 ± 3.99 points, indicating reduced sleep quality in the study group. After completing the eight-week daily therapy, this value decreased significantly to 4.13 ± 3.33 points (*p* < 0.001). The mean change was nearly 3 points, which is both statistically and clinically significant. These results suggest that the intervention contributed to a significant improvement in sleep quality in alcohol-dependent individuals, and a significant proportion of the participants moved from the “poor sleepers” group (PSQI > 5 points) to the “good sleepers” group (Figure 2).

The results are summarized in Table 1.

Post hoc power analysis showed high statistical power to detect observed pre-post differences, with values of 0.795 for the AIS and 0.998 for the PSQI, indicating that the study had sufficient power to detect changes in insomnia severity and sleep quality.

Analysis of Individual PSQI Components
  - Subjective sleep quality: participants rated their sleep as significantly better after completing therapy (mean score decreased from 1.52 to 0.84; *p* < 0.001). This change indicates an improved subjective feeling of rest and recovery after sleep.
  - Sleep latency: a reduction in the time it took to fall asleep was observed (from 2.54 to 1.90; *p* < 0.001). This is one of the most significant improvements and is clinically significant, as prolonged sleep latency is a typical symptom of insomnia.
  - Sleep duration: the average number of hours of sleep increased, as reflected in a decrease in scores from 0.86 to 0.42 (*p* < 0.001). This indicates an increase in sleep duration in the study group.
  - Sleep efficiency: this indicator improved significantly (from 1.00 to 0.44 points; *p* < 0.001), meaning that patients spent more time in bed actually sleeping.
  - Sleep disturbances: no significant changes were found (1.04 vs. 1.00 points). This may suggest that some sleep disturbances persist regardless of therapeutic intervention.
  - Sleep medication use: no significant differences were found (0.44 vs. 0.30 points; *p* = 0.429). This indicates that the improved sleep quality was a result of therapeutic interventions and not increased pharmacotherapy.
  - Daytime dysfunctions: this component decreased from 0.74 to 0.26 points (*p* = 0.001), reflecting improved daytime functioning and reduced fatigue and sleepiness.

## 4. Discussion

Given the prevalence of alcohol use disorder (AUD) in the Polish population and its impact on sleep disorders, which consequently impacts the functioning of individuals and society as a whole, an attempt was made to demonstrate the validity of day therapy for alcohol-dependent individuals to improve their quality of life. This study demonstrated that eight weeks of day therapy for alcohol-dependent individuals was associated with a significant and clinically meaningful reduction in insomnia severity (AIS) and improvement in sleep quality (PSQI). The mean of total PSQI score decreased from a level clearly exceeding the clinical threshold for poor sleep quality to a value close to normal, and the greatest improvements were observed in sleep latency, subjective sleep quality, and sleep efficiency, with stable (unchanged) use of sleep medication. This result suggests that the improvement was primarily due to therapeutic interventions rather than intensified pharmacotherapy and indicates the key role of psychotherapy, abstinence, and environmental interventions in sleep regulation in AUD. However, because the study did not include a control group, it is not possible to separate the effects of abstinence and spontaneous recovery from the specific effects of the day therapy program; therefore, this study should be interpreted as a before-and-after case series describing changes in sleep during routine inpatient day therapy treatment, rather than as evidence of causal treatment efficacy. This interpretation is supported by previous interventional studies using validated self-report measures such as the PSQI and ISI, which have shown that clinically significant sleep improvements can occur without increasing the use of sleep medications. For example, De Simone et al. demonstrated significant improvements in sleep quality and insomnia severity following a nonpharmacological intervention, underscoring the utility of these tools in capturing therapeutic sleep changes independent of medication effects [24]. The “integrative approach” described in this article is not specific to the examined health center, but rather reflects the currently dominant way of organizing addiction psychotherapy in Poland. Many public and private addiction treatment facilities utilize models combining elements of cognitive behavioral therapy, a humanistic-experiential approach, and motivational interviewing. This stems from both clinical recommendations and the practical needs of working with a patient population with diverse deficits, motivational levels, and co-occurring mental health issues. Integrating techniques and interventions from various approaches is common practice in the Polish addiction treatment system, not a proprietary solution of a single team. Therefore, the obtained results can be broadly generalized to other centers offering treatment based on a similar, unified integrative model in Poland. At the same time, we recognize that local organizational and staffing differences may influence the details of program implementation.

The results obtained in our study are consistent with previous reports indicating that sleep disturbances are one of the most common and persistent complaints among alcohol-dependent individuals. Epidemiological data from large population-based samples show that sleep problems—especially difficulty falling asleep, staying asleep, and the use of sleep medications—are significantly associated with the risk of problematic alcohol use, regardless of gender and race [25]. These findings are consistent with those obtained in our study: individuals with AUD who began treatment indeed demonstrated very high levels of sleep disturbances, as confirmed by our results (PSQI = 7.12; AIS = 9.09 at the beginning of treatment).

Intervention studies, including randomized clinical trials of CBT-I in patients with AUD, confirm that targeted insomnia treatment effectively improves sleep parameters and reduces daytime dysfunction, although it does not always affect the frequency of heavy drinking episodes [26]. Clinical trials of CBT-I in individuals with alcohol dependence confirm that targeted insomnia therapy significantly improves sleep quality and daytime functioning [27]. Although its impact on relapse rates and drinking intensity is less clear, these results suggest that sleep disorders should be considered a separate therapeutic target. Our observations complement these data by demonstrating that daytime therapy alone, combined with continued abstinence, can lead to significant improvements in sleep quality, even without specialized insomnia-focused interventions. This suggests that addiction treatment may partially function as a sleep intervention, but if symptoms persist, it is worth considering implementing additional methods, such as CBT-I. From a clinical perspective, these results indicate that routine monitoring of sleep quality during daytime inpatient treatment may help identify patients who could benefit from targeted interventions for insomnia.

Similar conclusions were reached by the authors of a randomized study, who found that participants in CBTI-AD, a cognitive-behavioral therapy for insomnia, showed greater improvement in one of the main sleep diary outcomes, i.e., sleep efficiency and selected daytime symptoms, including assessments of insomnia severity and fatigue, and were more likely to be classified as sleep treatment responders than participants in the control group [28]. The authors of a study conducted on a group of young adults also point to the benefits of CBT-I in people who actively drink alcohol [29]. The authors of another study additionally recommend the introduction of sleep hygiene education [13].

The AIS results confirm a reduction in the severity of insomnia symptoms; this scale has well-established content and criterion validity, and cutoffs of approximately ≥6–8 are commonly used for clinical classification. Our results are within the range consistently indicating improvement in this regard. Studies by other authors also support these findings [30].

The results of another study suggest the need for further interventions and larger-scale studies. The authors conducted a study examining the impact of alcohol craving on insomnia. The results showed that increased craving was associated with the insomnia symptom of difficulty falling asleep [31].

Cross-sectional data are also available in the literature, confirming a significant association between increased alcohol use and poorer sleep quality. Park et al. [32] in a study conducted in Korea using the PSQI-K (the Korean version of the PSQI) and the AUDIT-KR, demonstrated that in men, higher levels of alcohol consumption were significantly correlated with the PSQI total score and the components: subjective sleep quality, sleep duration, and sleep disturbances. In women, however, an association was observed only with daytime dysfunctions. These results support our observations that sleep disturbances intensify with increasing alcohol consumption in the AUD population. At the same time, they highlight the limitations of previous studies, which were primarily cross-sectional and did not include assessment of changes over time. Our before and after case series data therefore represent an important addition to existing knowledge, demonstrating that daytime therapy can significantly improve sleep quality.

Our study should be considered a preliminary step towards understanding sleep dynamics during addiction treatment. Future studies with a controlled group, longer follow-up, and the use of objective measurement methods (polysomnography, actigraphy) are necessary. Another important direction is to examine whether early sleep improvement (e.g., a decrease in PSQI during the first weeks of treatment) contributes to better treatment efficacy and a lower risk of relapse to alcohol use.

### Limitations

There are certain limitations to the conclusions drawn from this study. The lack of a control group is a major limitation of this study. Because all participants abstained from alcohol and received psychosocial support during the follow-up period, it is impossible to determine the extent to which the observed improvements in sleep quality and insomnia severity were due to the day therapy program itself, or to what extent to the effects of abstinence, stabilization of daily activities, or natural recovery during early remission. Because the therapeutic interventions simultaneously incorporate elements of cognitive behavioral therapy, a humanistic approach, and motivational interviewing, it is difficult to clearly determine which elements are responsible for the observed effects. Improvement may result from cognitive restructuring, increased motivation, a therapeutic relationship, or a synergy of these factors. Therefore, the study evaluates the effectiveness of the treatment package, not individual techniques. Furthermore, the lack of subgroup analyses and a control group limits the ability to determine which patients benefit most and how day therapy compares to other treatment methods. Another limitation was the exclusive use of self-report methods—the AIS and PSQI are reliable tools, but based on patient self-reports. The lack of objective sleep measurements (e.g., polysomnography, actigraphy) may limit the accuracy of conclusions, especially in the addicted population, where subjective sleep perception can be impaired. Another limiting factor was the observation time—the study covered only eight weeks. The lack of long-term data prevents assessment of the durability of the therapy’s effects, which may have limited our study. Furthermore, the homogeneity of the sample—the participants came from a single treatment center in a single region of Poland, which limits the generalization of the results to other patient populations. Another limiting factor was the possible expectation effect—the improved sleep quality could have been partially related to a sense of participation in therapy and greater support, rather than solely to an actual physiological change in sleep. Despite these limitations, the obtained results provide significant evidence of the effectiveness of daytime therapy in improving sleep quality in alcohol-dependent individuals and serve as a starting point for further research using objective sleep measurement methods and a control group.

## 5. Conclusions

1. Daytime therapy conducted over an eight-week period significantly reduces the severity of insomnia symptoms in alcohol-dependent individuals, as evidenced by a decrease in the mean AIS score of over 40%.
2. The PSQI total score decreased significantly, from values clearly indicating “poor sleep quality” to a cutoff level, suggesting improvement in the study population.
3. The greatest improvements were observed in sleep latency, subjective sleep quality, and sleep efficiency, while the use of sleep-inductive drugs and the frequency of sleep disturbances did not change significantly.
4. The results confirm that therapeutic interventions, regardless of pharmacotherapy, can effectively improve sleep quality in the alcohol-dependent population.
5. These results support the need to include routine assessment of sleep quality and insomnia severity in addiction treatment in day hospitals, as improved sleep may be an important indicator of early recovery and treatment engagement.

## Figures and Tables

**Table 1:** PSQI component analysis before and after therapy.

Component PSQI	Before Therapy (M ± SD)	Median [Q1–Q3]	After Therapy (M ± SD)	Median [Q1–Q3]	*p*
Subjective sleep quality	1.52 ± 0.89	2 [1–2]	0.84 ± 0.72	1 [0–1]	<0.001
Sleep latency	2.54 ± 0.74	3 [2–3]	1.90 ± 0.77	2 [1–2]	<0.001
Sleep time	0.86 ± 1.02	1 [0–2]	0.42 ± 0.73	0 [0–1]	<0.001
Sleep efficiency	1.00 ± 1.11	1 [0–2]	0.44 ± 0.68	0 [0–1]	<0.001
Sleep disorders	1.04 ± 0.44	1 [1–1]	1.00 ± 0.44	1 [1–1]	-
Sleeping medication	0.44 ± 1.18	0 [0–0]	0.30 ± 1.11	0 [0–0]	0.429
Daily disfunctions	0.74 ± 0.72	1 [0–1]	0.26 ± 0.45	0 [0–0]	0.001

Legend: M—median; SD—standard deviation; *p*—statistical significance.

## Data Availability

The data analysed during the current study are available from the authors.

## Supplementary Materials

The following supporting information can be downloaded at: https://www.mdpi.com/article/10.3390/jcm15041400/s1, File S1:CARE Checklist of information to include when writing a case report.

## Abbreviations

The following abbreviations are used in this manuscript:

AUD	Alcohol Use Disorders
AIS	Athens Insomnia Scale
PSQI	Pittsburgh Sleep Quality Questionnaire
DSM-V	Diagnostic and Statistical Manual of Mental Disorders
CBI-I	Cognitive-Behavioral Therapy
CBTI-AD	Cognitive Behavioral Therapy for Insomnia in Alcohol Dependence
PSQI-K	Pittsburgh Sleep Quality Index—Korean version
AUDIT-KR	Alcohol Use Disorders Identification Test—Korean version
