# Supplementary material for: Changes in Sleep Quality and Insomnia Severity After Day Therapy in Patients with Alcohol Dependence: A Before-and-After Case Series
Source: J Clin Med. 2026 Feb 11;15(4):1400. doi: 10.3390/jcm15041400 (PMC12942477; doi:10.3390/jcm15041400)
Supplement: Supplementary file 1 [file jcm-15-01400-s001.zip › jcm-4081956-supplementary.pdf]

CARE case report guidelines

CARE Checklist of information to include when writing a case report

(cc) BY-NC-ND

Topic	Item No	Checklist item description	Reported on Page Number/Line Number	Reported on Section/Paragraph
Title	1	The diagnosis or intervention of primary focus followed by the words “case report”	Line 2-4	Title
Key Words	2	2 to 5 key words that identify diagnoses or interventions in this case report, including "case report"	Line 38	Keywords
Abstract (Structured summary)	3a	Background: state what is known and unknown; why the case report is unique and what it adds to existing literature.	Line 18-24	Abstract
	3b	Case Description: describe the patient’s demographic details, main symptoms, history, important clinical findings, the main diagnosis, interventions, outcomes and follow-ups.	Line 25-35	Abstract
	3c	Conclusions: summarize the main take-away lesson, clinical impact and potential implications.	Line 35-37	Abstract
Introduction	4	One or two paragraphs summarizing why this case is unique **(may include references)**	Line 87-91	Introduction
Patient Information	5a	De-identified patient specific information	Line 108-115	Materials and Methods/Particip ants
	5b	Primary concerns and symptoms of the patient	Line 102-103	Materials and Methods/Setting and Ethical Consideration
	5c	Medical, family, and psycho-social history including relevant genetic information	Line 108-115	Materials and Methods/Particip ants
	5d	Relevant past interventions with outcomes	Not applicable	Not applicable
Clinical Findings	6	Describe significant physical examination (PE) and important clinical findings	Line 198-201<br>Line 206-210<br>Line 225-226	Results

Timeline	7	Historical and current information from this episode of care organized as a timeline	**Line 101<br>Line 118<br>Line 98<br>Line 108-109**	**Materials and Methods/Setting and Ethical Consideration and Participants and Study Desing**
Diagnostic Assessment	8a	Diagnostic testing (such as PE, laboratory testing, imaging, surveys).	**Line 147-148**	**Materials and Methods/Data Sources**
	8b	Diagnostic challenges (such as access to testing, financial, or cultural)	**Not applicable**	**Not applicable**
	8c	Diagnosis (including other diagnoses considered)	**Line 103**	**Materials and Methods/Setting and Ethical Consideration**
	8d	Prognosis (such as staging in oncology) where applicable	**Not applicable**	**Not applicable**
Therapeutic Intervention	9a	Types of therapeutic intervention (such as pharmacologic, surgical, preventive, self-care)	**Line 116-145**	**Materials and Methods/Particip ants**
	9b	Administration of therapeutic intervention (such as dosage, strength, duration)	**Line 116-145**	**Materials and Methods/Particip ants**
	9c	Changes in therapeutic intervention (with rationale)	**Not applicable**	**Not applicable**

Follow-up and Outcomes	10a	Clinician and patient-assessed outcomes (if available)	**Line 194-238**	**Results**
	10b	Important follow-up diagnostic and other test results	**Line 196-197<br>Line 205-206<br>Line 215-216<br>Line 220-238**	**Results**
	10c	Intervention adherence and tolerability (How was this assessed?)	**Not applicable**	**Not applicable**
	10d	Adverse and unanticipated events	**Not applicable**	**Not applicable**
Discussion	11a	A scientific discussion of the strengths AND limitations associated with this case report	**Line 243-249<br>Line 252-256<br>Line 275-277<br>Line 291-293<br>Line 325-333<br>Line 335-363**	**Discussion and Limitations**
	11b	Discussion of the relevant medical literature **with references**	**Line 240-333**	**Discussion**
	11c	The scientific rationale for any conclusions (including assessment of possible causes)	**Line 240-333**	**Discussion**
	11d	The primary “take-away” lessons of this case report (without references) in a one paragraph conclusion	**Line 365-377**	**Conclusions**
Patient Perspective	12	The patient should share their perspective in one to two paragraphs on the treatment(s) they received	**Not applicable**	**Not applicable**
Informed Consent	13	Did the patient give informed consent? Please provide if requested	☐ **Yes X**	**No** ☐
